# Ion Channels in Glioblastoma

**DOI:** 10.5402/2011/590249

**Published:** 2011-11-29

**Authors:** Remco J. Molenaar

**Affiliations:** Department of Cell Biology and Histology, Academic Medical Center, University of Amsterdam, Meibergdreef 15, 1105 AZ Amsterdam, The Netherlands

## Abstract

Glioblastoma is the most common primary brain tumor with the most dismal prognosis. It is characterized by extensive invasion, migration, and angiogenesis. Median survival is only 15 months due to this behavior, rendering focal surgical resection ineffective and adequate radiotherapy impossible. At this moment, several ion channels have been implicated in glioblastoma proliferation, migration, and invasion. This paper summarizes studies on potassium, sodium, chloride, and calcium channels of glioblastoma. It provides an up-to-date overview of the literature that could ultimately lead to new therapeutic targets.

## 1. Introduction

Glioblastoma (astrocytomas, WHO grade IV) is the most aggressive primary brain tumor. With an incidence of 3.5 per 100,000 people per year, it may affect children, adults, and elderly. However, it preferentially affects adults between 45 and 75 years of age [1]. 

Glioblastomas can either present themselves as primary glioblastomas (95%), which manifest *de novo *and lack precursor tumors, or secondary glioblastomas. These tumors have progressed from less malignant glioma [2]. 

Surgery is the initial intervention when a patient has been diagnosed with a brain tumor. This is needed to obtain a histological diagnosis and reduces the space-occupying effect of the tumor. However, in glioblastoma, surgery is of limited therapeutic value, as complete resection is impossible due to the extensive invasive, and migratory behavior of glioblastoma cells. This renders radiotherapy ineffective as well. The current treatment is concomitant administration of temozolomide and radiotherapy. However, median survival is only 15 months [3].

The understanding of molecular alterations in signaling pathways and the consequent pathology in glioblastoma has greatly increased in the last years due to the availability of new techniques, such as genome-wide sequencing. One of the pathways that are frequently affected in glioblastoma includes channels involved in transport of sodium, potassium, and calcium ions [4]. The present paper provides an overview of the current evidence of the involvement of these ion channels in glioblastoma in terms of gliomagenesis, glioma progression, and their effect on prognosis. Because of the progression of lower-grade glioma to glioblastoma, the involvement of ion channels in high-grade glioma is discussed as well. Finally, the application of these insights is discussed in the light of future prospects for experimental and clinical practice. 

## 2. Ion Channels and Glioblastoma

Glial cells express a variety of ion channels [5]. Recently, genome-wide analyses of glioblastoma became available. A survey of the coding sequence of 20,661 genes in glioblastoma genomes has implicated many new gene alterations. One cluster of mutated genes reported was that of ion channel genes. Of the 555 genes involved in potassium, sodium, chloride, calcium and other ion transport, 55 mutations were detected to affect 90% of the glioblastoma samples studied [4].

Ion channels are thought to facilitate progression through cell cycle checkpoints and thereby are required for cell proliferation. This process most probably occurs via modulation of the resting membrane potential. For example, progression through the G_1_/S checkpoint is correlated with increased potassium K^+^ channel activity and momentary hyperpolarization [6]. To illustrate this, iberiotoxin, a pharmacological inhibitor of big conductance K^+^ channels, arrests glioma cells in S phase of the cell cycle [7]. On the other hand, transient depolarization facilitated by Cl^−^ channels is observed at the G_2_/M checkpoint [6]. It is for these reasons that uncontrolled ion channel activity can contribute to oncogenesis.

**Figure 1:**
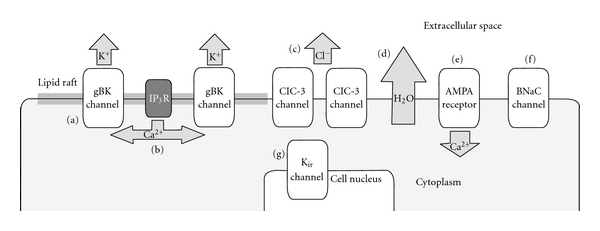
gBK channels (a) facilitate an increased outwardly K^+^ current. Ca^2+^ input for gBK channels is provided by IP_3_R (b). ClC-3 channels facilitate an increased Cl^−^ outwardly current. (a) and (c) facilitate increased H_2_O movement through osmosis (d) over the plasma membrane. Glioblastoma AMPA receptors (e) lack the GluR2 subunit and therefore have increased Ca^2+^ permeability. Amiloride-sensitive BNaC channels (f) are expressed in glioblastoma. K_ir_ channels are mislocalized to the cell nucleus (g), diminishing inwardly rectifying K^+^ currents.

As stated before, the prognosis of glioblastoma is abysmal due to its invasive migration, which renders surgical resection ineffective. Ion channels may contribute to this invasion and migration. They influence shape and volume of cancer cells by affecting ion and water transport over the plasma membrane. Ion channels thereby facilitate invasive migration through the sinuous extracellular space of brain tissue [8–13] (Figure 1). In addition, ion channels may be functionally involved in proliferation [7, 14, 15]. It is for these reasons that ion channels may contribute to the malignant behavior of glioblastoma cells. Therefore, ion channels may be novel therapeutic targets in the treatment of glioblastoma.

### 2.1. Potassium Channels

#### 2.1.1. Ca^2+^-Activated K^+^ Channels

Ca^2+^-activated K^+^ channels facilitate outwardly rectifying potassium currents and respond to Ca^2+^ concentrations. Increases in the intracellular [Ca^2+^] shift the voltage dependence of Ca^2+^-activated K^+^ channels to more negative potentials.

One of these channels, big conductance K^+^ channels or BK channels, is widely expressed in excitable and non-excitable cells. BK channels respond to both membrane voltage potentials and intracellular [Ca^2+^] [16].

Specific overexpression of BK channels has been observed in biopsies of patients with malignant gliomas, compared with nonmalignant human cortical tissues. In addition, expression levels correlate positively with the malignancy grade of the tumor [16]. Lastly, BK currents in glioma cells are more sensitive to intracellular [Ca^2+^] compared to BK channels in healthy glial cells [17, 18].

BK channels can express a variety of electrophysiological properties, which is due to alternative splicing of their *α*-subunits. The increased sensitivity to intracellular [Ca^2+^] is found in a novel splice isoform of *hSlo*, the gene that encodes the *α*-subunits. This BK channel isoform has exclusively been observed to be expressed in glioma. In addition, glioma most likely only expresses this new isoform, as the classical BK channel has not been found in gliomas yet. These findings led to the term glioma BK (gBK) channel (Figure 1).

gBK channels have been suggested as a candidate channel for providing the electrochemical driving force for ion movement needed for the release of cytoplasmic water and cell shrinkage which in turn facilitates the extensive migrating behavior of glioblastoma cells. First of all, the effect of menthol was studied, an agonist of transient receptor potential melastatin 8 (TPRM8) ion channels, which increases intracellular [Ca^2+^], which in turn activates gBK channels. Menthol stimulated glioma cell migration [11, 12]. In addition, administration of paxilline and tetraethylammonium, both gBK channel inhibitors, inhibited migration [11, 12]. These findings make a role of gBK channels in the migration of glioblastoma cells probable. However, the effect of gBK channel knockdown has yet to be investigated, leaving room for doubt.

In contrast to the better studied role that gBK channels have in glioblastoma cell invasive migration, there is discussion whether or not gBK channels contribute to proliferation. Some studies have implicated gBK channels in the proliferation of glioblastoma cells. Glioblastoma cells were exposed to pharmacological inhibitors of gBK channels, such as iberiotoxin, paxilline, tetraethylammonium, and penitrem A. After 3 to 5 days, growth inhibition and even tumor shrinkage were observed *in vitro *[7, 14, 15]. In contrast, more recent literature contradicts these findings and suggests that gBK channels are not required for proliferation or even have antitumorigenic properties. It has been found that pharmacological inhibitors suppress glioblastoma cell growth at concentrations far higher than concentrations that were sufficient to inhibit gBK channel activity. Low concentrations that were sufficient to inhibit these channels did not affect glioblastoma cell growth *in vitro*. In addition, downregulation of gBK channels using gene-specific siRNAs reduced K^+^ current densities, but caused no changes in proliferation [19]. This argues against a critical role for gBK in glioblastoma proliferation.

Inositol 1,4,5-triphosphate receptors (IP_3_Rs) may provide Ca^2+^ for gBK channels (Figure 1). These receptors are localized close to and are linked with gBK channels in dedicated plasma membrane domains called lipid rafts. Disruption of these lipid rafts with methyl-*β*-cyclodextrin disturbs the connection between gBK channels and Ca^2+^. This disturbance was restored by inclusion of Ca^2+^ in the pipette solution during the whole cell patch-clamp experiments. This suggests that the disturbance was not caused by destruction or calcium desensitization of the gBK channels [20].

#### 2.1.2. Inwardly Rectifying K^+^ Channels

Glioma cells both *in vitro* and *in situ* are characterized by depolarized resting membrane potentials of about −20 to −40 mV [21]. In addition, they express increased outwardly rectifying K^+^ currents [16]. This contrasts the very negative resting membrane (−80 to −90 mV) and large inwardly rectifying K^+^ currents that characterize normal glial cells [22]. These findings have led to the assumption that glioma cells express a decreased density of inwardly rectifying K^+^ channels (K_ir_) compared to normal brain tissue.

However, western blot analysis of D54 glioblastoma cell lines showed only slightly lowered expression of K_ir_2.1, normal expression of K_ir_4.1, and increased expression of K_ir_2.3 and K_ir_3.1 as compared to normal astrocytes, while electrophysiological recording found no K_ir_ current. Immunocytochemistry placed suspicion on mislocalization of K_ir_ channels in glioblastoma cells. Immunostaining of K_ir_2.3 and K_ir_4.1 predominantly labeled the nucleus of glioblastoma cells (Figure 1), while expression in normal astrocytes was diffuse over the plasma membrane [23]. The intracellular localization of K_ir_ channels may explain why glioblastoma cells express a depolarized resting membrane potential and decreased inwardly rectifying K^+^ current. 

K_ir_ channels in glial cells are specifically known for two functions: buffering of extracellular K^+^ and establishment of a very negative resting potential [24]. Glial cells perform K^+^ uptake from the extracellular space and redistribute K^+^ ions toward areas where the extracellular [K^+^] is lower. This process includes diffusion from cell to cell through gap junctions. Eventually, K^+^ is released into blood vessels. This K^+^ buffering by glial cells is essential for neuronal homeostasis, as elevated extracellular [K^+^] would depolarize neurons, preventing them from firing action potentials. K_ir_ channels in healthy glial cells seem to be fitted very well for this task, as they have a large open probability at resting potential. This means that at resting potential, a relatively high number of K_ir_ channels are open. In addition, with increasing extracellular [K^+^] their conductance increases as well, which makes them perfectly suited for the task of correcting an excess of K^+^ in the extracellular space [22]. Mislocalization of K_ir_ channels in glioblastoma cells (Figure 1) renders these cells insufficient for these tasks, resulting in accumulation of K^+^ in the extracellular space. This accumulation occurs in concurrence with epileptic seizures [25], although the underlying mechanism is not entirely clear. Peritumoral epileptic seizures are often seen in glioblastoma patients, perhaps facilitated by mislocalization of K_ir_ channels. On the other hand, it is likely that very few to no neurons survive around glioblastoma cells due to their invasive behavior. Moreover, increased concentrations of glutamate in gliomas have been reported [26]. These arguments, supported by the excitotoxic properties of high glutamate concentrations to neurons, render high glutamate release a more logical suspect of causing epileptical seizures than high [K^+^] in the extracellular space.

On the other hand, mislocalized K_ir_ channels can contribute indirectly to epileptic seizures as they fail in their function of establishing a very negative resting potential. At the depolarized resting potentials of −20 to −40 mV that characterize glioma cells [21], the Na^+^ gradient across the plasma membrane is diminished. As a consequence, Na^+^-dependent glutamate transporters become inactive, increasing the extracellular glutamate concentration and thereby possibly causing epileptic seizures [23].

#### 2.1.3. Ether À Go-Go K^+^ Channels

Other candidate channels possibly responsible for the depolarized resting membrane potential in glioblastoma cells are the ether à go-go 1 (EAG1) and ether à go-go related 1 (ERG1) channels. Expression of these channels is upregulated in glioblastoma. Depolarized resting membrane potential allows large hyperpolarizations, which provide a driving force for Ca^2+^ entry. Ca^2+^ is necessary for cell-cycle progression. In this way, EAG1 and ERG1 channels can contribute to gliomagenesis. 

Several studies have described expression levels of *hEAG1 *and *hERG1 *in glioblastoma, which encodes EAG1 and ERG1 channels, respectively. However, the results are contradictory. Patt et al. reported low expression of *hEAG1* and *hERG1* in 5 glioblastoma samples compared to healthy brain tissue. Expression was elevated in low-grade gliomas [27]. This contradicts with the hypothesis above. In contrast, Masi et al. found elevated expression of *hERG1* in 26 glioblastoma samples, supporting the hypothesis above [28]. The observation by Masi et al. is supported by other literature, reporting increased ERG1 mRNA expression, elevated protein levels and high densities of ERG1 channels in other tumors, such as colorectal cancer [29], endometrial adenocarcinoma [30], and myeloid leukemia [31].

ERG1 activity has also been reported to be correlated with induction of vascular endothelial growth factor (VEGF) secretion, thereby contributing to angiogenesis [28]. Primary glioblastomas are characterized by extensive neoangiogenesis [32]. 

### 2.2. Chloride Channels

#### 2.2.1. ClC Family Channels

Besides the gBK channel discussed earlier, the ClC-3 chloride channel is another important candidate channel to facilitate migrating behavior of glioblastoma cells [10]. ClC-3 channel expression was, together with that of ClC-2 and ClC-5, indeed high in glioblastoma cell lines and biopsy tissue compared to healthy glial cells. Whole cell patch-clamp recordings in combination with channel-specific antisense oligonucleotides showed that ClC-3 channels facilitated outwardly rectifying current. On the other hand, ClC-2 channels facilitated inwardly rectifying currents [33]. As a result of the relative overexpression of ClC-3 as compared to ClC-2 [33], the outwardly rectifying current may prevail over the inwardly rectifying current. As a consequence the glioblastoma cell depolarizes, which is needed to pass the G_2_/M checkpoint in the cell cycle [6].

In addition, the overexpression of both channels equips glioblastoma cells with an improved ability to transport Cl^−^. This may facilitate rapid changes in cell size and shape as glioblastoma cells invade through sinuous extracellular brain spaces [33].

Inhibition of ClC-3 channels using chlorotoxin indeed markedly but not completely inhibited glioblastoma cell invasion *in vitro*. The same effect was accomplished using ClC-3 siRNA knockdown. In addition, the nonspecific ClC-blocker 5-nitro-2-(3-phenylpropylamino) benzoic acid (NPPB) completely inhibited glioblastoma cell invasion [9]. Limitations of this study include the possibility that the glioblastoma cell was overdosed with NPPB and invasion was stopped by cytotoxic levels of NPPB. It is unclear whether concentrations of NPPB were just sufficient to block ClC channels. This doubt was also raised in studies where gBK channel inhibition correlated with proliferation [19]. Secondly, NPPB has also been reported to be a Ca^2+^-activated K^+^-channel inhibitor [34]. This fact may compromise the implication of ClC-3 in glioblastoma cell invasion.

ClC-3 is regulated through phosphorylation via Ca^2+^/calmodulin-dependent protein kinase II (CaMKII). CaMKII was infused intracellularly to D54 glioblastoma cells via a patch-clamp pipette, increasing Cl^−^ currents 3-fold. In addition, administration of autocamtide-2, a CaMKII-specific inhibitor, inhibited this current. To confirm the relation between ClC-3 and CaMKII, ClC-3 was shown to be knocked down after CaMKII modulation of Cl^−^ currents was lost. Furthermore, immunohistochemistry colocalized ClC-3 with CaMKII. Interestingly, inhibition of CaMKII in ClC-3-expressing cells reduced glioblastoma cell invasion to the same extent as direct inhibition of ClC-3 [35]. These findings suggest that CaMKII is a molecular link between intracellular [Ca^2+^] changes and ClC-3 conductance required for cell movement during invasive migration in glioblastoma.

### 2.3. Calcium Channels

Ca^2+^ is required by glioblastoma cells as a second messenger to support cell migration. Oscillatory changes in intracellular [Ca^2+^] that correlate with cell invasion and migration have been observed. It has been hypothesized that these changes in intracellular [Ca^2+^] activate ClC-3 channels through CaMKII. This in turn may initiate glioblastoma invasion [35, 36].

Glioblastoma cells express Ca^2+^ permeable alpha-amino-3-hydroxy-5-methyl-4-isoxazolepropionate (AMPA) glutamate receptors (Figure 1). These glutamate receptors have become Ca^2+^ permeable due to the lack of the GluR2 subunit as they have been assembled entirely of GluR1 and/or GluR4 subunits. AMPA receptors containing GluR2 subunits show little Ca^2+^ permeability, while those lacking GluR2 subunits exhibit high Ca^2+^ permeability due to a deformed pore with an aberrant size and polarity. Adenovirus-mediated transfer of the GluR2 cDNA decreased intracellular [Ca^2+^], inhibited cell migration and induced apoptosis. However, overexpression of Ca^2+^ permeable AMPA receptors increased migration and proliferation of glioblastoma cells *in vitro*. GluR2 was indeed not expressed in most glioblastoma surgical samples. These findings implicate Ca^2+^-permeable AMPA receptors in proliferation, migration and invasion of glioblastoma [8]. It can be hypothesized that this is due to increased intracellular [Ca^2+^], which in turn facilitates increased activity of gBK and ClC-3 channels.

The same theory applies to a study in which inhibition of IP_3_R subtype 3 (IP_3_R3) was achieved using caffeine. Inhibition of IP_3_R3 led to decreased intracellular [Ca^2+^]. This was associated with inhibited migration of glioblastoma cells *in vitro*. In addition, an increase of mean survival was observed after caffeine was administered to a mouse xenograft model of glioblastoma. These mice had a 6 *μ*g/mL serum caffeine concentration. This is approximately the same concentration in people that drink two to five cups of coffee a day. These findings suggest that IP_3_R3 can serve as a possible therapeutic target [13]. It would be interesting to investigate whether large cohort studies can associate coffee consumption with a lower incidence of glioblastoma or prolonged survival. However, such information is not available yet. With a yearly glioblastoma incidence of 3.5 per 100,000 people, study sizes are probably not large enough to show such results. The effect of caffeine on the survival of glioblastoma mouse models is interesting as caffeine is regarded as “not classifiable as to its carcinogenicity to humans” by the WHO, meaning that there is contradictory evidence about its carcinogenic hazard [37].

The Ca^2+^-permeable transient receptor potential canonical channel protein 6 (TRPC6) has been implicated in glioma proliferation. TRPC6 is overexpressed in human glioma cells, and inhibition suppressed intracellular [Ca^2+^] and cell growth and induced cell cycle arrest at the G_2_/M phase. In mouse models with xenografted human tumors, inhibition of TRPC6 reduced tumor volume and increased mean survival [38].

### 2.4. Sodium Channels

In contrast to mutations found in potassium and calcium ion channels, sodium channel mutations correlated with shorter survival in a univariate analysis [39]. The authors reported that all samples with *IDH1* mutations did not have any sodium channel mutations. However, this association was not significant. This may be due to the small sample size of the study (21 patients).

*IDH1* encodes for an enzyme that functions at a crossroads of cellular metabolism. Mutations in *IDH1* have been identified to be associated with a specific subgroup of glioblastoma patients who are younger and have a prolonged survival [4, 40]. After correction for *IDH1*, the difference in survival between patients with mutated and unmutated sodium channels dropped to nonsignificant levels. Further research is needed to investigate the association between mutations in *IDH1* and sodium channels and whether the effect of sodium channel mutations on survival is independent of *IDH1* mutation status.

Furthermore, among the patients with mutations in sodium channels, the mutations were scattered over the different genes. All 14 sodium channel genes were mutated only once among the 21 patients except for *SCN9A*, which was mutated twice. In addition, of the 12 patients with sodium channel mutations, only 2 had mutations in more than one gene [39]. This could suggest a similar function, and therefore mutual exclusivity among these mutations. This is supported by the fact that of the 14 studied genes, 12 genes were from the *SCN* or *SLC* subset classes with 5 and 7 genes, respectively. A similar scattering among the studied genes and clustering in gene families was observed in potassium and calcium channel mutation analyses [39]. 

Moreover, the effect of sodium channel inhibitors on glioblastoma cell growth was studied. Digoxin and ouabain were administered to 2 glioblastoma cell lines *in vitro*. Both drugs showed antiproliferative effects and toxicity against the cell lines. Furthermore, cells treated showed an apoptotic phenotype under the light microscope [39]. These findings are supported by the fact that the antiproliferative effect in cancer of cardiac glycosides is well known [41, 42]. Furthermore, they may be neuroprotective [43]. The underlying mechanism of these side effects has not been clarified yet, although inhibition of sodium channels in brain tissue could be the cause of this.

Another study observed an inward, amiloride-sensitive Na^+^ current in glioblastoma cell lines and tumor samples. These currents were not observed in normal astrocytes or low-grade astrocytomas. Currently, brain Na^+^ channels (BNaCs) are the only amiloride-sensitive Na^+^ channels identified in the brain. PCR analyses indeed demonstrated the presence of BNaC mRNA in these tumors [44].

Finally, the effect of Psalmotoxin 1, an inhibitor of cation currents mediated by acid-sensing ion channels, was studied using the whole-cell patch-clamp technique. This toxin inhibited Na^+^ currents in glioblastoma cell lines and human glioblastoma samples, but not in normal human astrocytes [45]. Since this effect can only be measured using electrophysiological experiments, the diagnostic value seems low. However, if the Na^+^ current facilitated by acid-sensing ion channels proves essential for glioblastoma cells *in vivo*, inhibition of Psalmotoxin may serve as a possible future therapy.

## 3. Future Therapeutic Targets

### 3.1. Ion Channels

Given the important role that gBK and ClC-3 channels are thought to have in glioma invasion and migration, these channels may render a promising therapeutic target to render glioblastoma less aggressive. However, even if *in vivo* inhibition of gBK and ClC-3 channels can inhibit glioblastoma invasion and migration, these future therapies probably have to be administered at an early stage in order to make a difference in the treatability by resection and radiotherapy.

Taken the central role of intracellular [Ca^2+^] in ClC-3 and gBK channel activity into account, influencing glioblastoma Ca^2+^ homeostasis may be a target of future therapies. This can be accomplished by inhibition of Ca^2+^-permeable AMPA receptors, for example by adenovirus-mediated transfer of GluR2 cDNA [8]. Another possible therapy is inhibition of IP_3_R3 using caffeine [13]. However, the carcinogenic hazard of caffeine is not clear yet [37]. On the other hand, Ca^2+^ coupling to ClC-3 and gBK channels can be disturbed by inhibiting CaMKII [35] or lipid rafts, [20] respectively. In addition, inhibition of TRPC6 has shown promising results both *in vitro* and *in vivo* in mouse models.

The finding that glioma cells' depolarized resting membrane potential and their inability to maintain K^+^, Na^+^ and glutamate homeostasis are caused by mislocalization of K_ir_ channels suggests that these channels function at a crossroads of cellular homeostasis and basic electrophysiological functions in glioma cells [23]. Correction of this mislocalization could therefore serve as a possible therapeutic target. However, the underlying mechanism of this mislocalization is currently unknown, making therapy uncertain in the near future.

Recently, the antiproliferative effect of cardiac glycosides on glioblastoma cell growth *in vitro* was studied. Digoxin and ouabain proved useful inhibitors of cell proliferation [39]. However, concentrations that provide an anticancer effect are high and induce severe cardiovascular side effects. Therefore, their development as anticancer agents has been limited thus far. Chemical modification is needed to increase affinity for tumor sodium channels and decrease affinity for cardiac sodium channels [42].

### 3.2. Chemotherapeutic Agents

Tetrandrine is an inhibitor of gBK channels. It therefore may inhibit the extensive invasion of glioblastoma cells. Moreover, tetrandrine has cytotoxic effects. Furthermore, it exacerbates radiation-induced cell-cycle perturbation, thereby inducing apoptosis and radiosensitization in glioblastoma cells. In addition, it has antiangiogenic effects. These capabilities render it a possible useful therapy to treat glioblastomas, especially combined with radiotherapy or other chemotherapeutic agents [46]. The combination of classical cytotoxic, apoptotic, radiosensitization and antiangiogenic effects with inhibition of gBK channels is promising. However, the effect of tetrandrine has thus far only been studied in rats [47]. Furthermore, tetrandrines' large arsenal of possible therapeutic targets in gliomas may impede to find an optimal dosage.

The blood-brain tumor barrier is an important hurdle to overcome in glioblastoma treatment. Temozolomide, a chemotherapeutic agent that was discussed earlier, is currently, together with radiotherapy, the golden standard in glioblastoma treatment. However, temozolomide crosses the blood-brain tumor barrier insufficiently to have a significant impact on patient survival. The same accounts for trastuzumab, which may be especially effective in a distinct glioblastoma subgroup (neural, as discussed by Verhaak et al. [48]) when combined with temozolomide. Therefore, both drugs were coinfused with minoxidil sulfate, an ATP-sensitive potassium channel (K_ATP_) activator. In mice, this indeed resulted in improved selective drug delivery to glioblastoma. The underlying mechanism is not completely understood, but it involves formation of brain vascular endothelial transcytotic vesicles to facilitate absorption of the drug [49]. 

### 3.3. Limitations of Cell Lines

Currently, most research in the field of ion channels in glioma is conducted on glioma cell lines. However, several experiments have shown that established glioblastoma cell lines resemble glioblastomas in patients very poorly when compared at the level of DNA alterations or gene expression profiles [50]. With this in mind, results from experiments conducted with cell lines should always be put into the right context. *In vitro* studies in human glioblastoma samples or *in vivo* studies in animal xenograft models remain needed.

## 4. Conclusion

At this moment, we have increased our understanding of the molecular mechanisms involving ion channels underlying the invasive migration of glioblastoma. However, in contrast to many other forms of cancer and considering the genetic research in glioblastoma, consequences for treatment are lagging behind. Under the shadow of the large and extensive research on genetic alterations and its effects on therapy responses in glioblastoma [51], it is doubtful whether any therapies involving ion channels will ever see the light. Our current understanding of ion channels in glioblastoma will most probably lead to drugs that can be given concomitantly with chemotherapeutic agents to increase their effectivity, such as the discussed coinfusion with minoxidil sulfate. In addition, the current knowledge of the involvement of BK channels, ClC-3 channels and intracellular [Ca^2+^] homeostasis in glioblastoma invasion and migration would justify the first steps in drug development targeting these aspects.
